# Trends of Opioid Use Disorder and Associated Factors in Hospitalized Patients With Arthritis

**DOI:** 10.7759/cureus.10203

**Published:** 2020-09-02

**Authors:** Adeolu O Oladunjoye, Olubunmi O Oladunjoye, Jean Gauvin, Maria Ruiza Yee, Eduardo D Espiridion

**Affiliations:** 1 Medical Critical Care, Boston Children's Hospital; Harvard Medical School, Boston, USA; 2 Psychiatry, Reading Hospital Tower Health, West Reading, USA; 3 Internal Medicine, Reading Hospital Tower Health, West Reading, USA; 4 Psychiatry, Philadelphia College of Osteopathic Medicine, Philadelphia, USA; 5 Psychiatry, Drexel University College of Medicine, Philadelphia, USA; 6 Psychiatry, West Virginia School of Osteopathic Medicine, Lewisburg, USA; 7 Psychiatry, West Virginia University School of Medicine, Martinsburg, USA

**Keywords:** opioid use disorders, hospitalization, opioid medication, opioid overdose, arthritis

## Abstract

Introduction

Opioid use was primarily limited to acute pain, postsurgical care, and end of life care setting but now is the most prescribed medication for chronic pain. Arthritis is a chronic disease associated with chronic pain. Given limited options for pain relief in the patient population, these patients are often prescribed opioids and are at increased risk of opioid use disorder (OUD). Therefore, our study aimed to identify factors associated with OUD in patients with arthritis.

Methods

We analyzed hospitalized adult patients with arthritis with and without OUD using discharge data from National Inpatient Sample (NIS) over five years from January 1, 2010, to December 31, 2014. We looked at trends of OUD in hospitalized patients with arthritis and compared demographic and clinical characteristics of patients with and without OUD using Student’s t-test and chi-square test. Multivariate analysis was also used to adjust for variables.

Results

A total of 21,396,252 arthritis hospitalizations were identified during the five-year study period among which 227,608 had OUD. The prevalence of OUD in arthritis hospitalization increased over the five-year period by 43%. After adjusting for other variables, mental health (OR 2.50 (2.43-2.58)), and substance use (OR 6.39 (6.14-6.66)) disorders were associated with increased odds of OUD.

Conclusion

The prevalence of OUD among patients with arthritis increased over the five-year study period. Mental health and substance use disorders were associated with increased odds of OUD. More studies are needed to explore alternative pain management options for arthritis patients particularly in those with mental health and substance use disorders.

## Introduction

Opioid use disorder (OUD) is described by the Diagnostic and Statistical Manual of Mental Disorders, 5th Edition as a problematic pattern of opioid use leading to significant problems or distress [1]. Opioid use was primarily limited to acute pain, post-surgical care and end of life care setting, but is now being increasingly used for the treatment of chronic pain with more than 100 million Americans now receiving treatment for chronic pain [2]. Opioids are now the most commonly prescribed medication for chronic pain. Recently, there are questions regarding their safety and efficacy in chronic pain treatment when not related to cancer or palliative care [3]. The recent trend of OUD, the so-called third wave, arose in 2013 as a result of the surge in prescription or illicitly-manufactured synthetic opioids, specifically tramadol, and fentanyl made available to people presenting with pain in the hospital [1]. This has led to a quadrupling of opioid-related deaths since the 2000s [4].

About 259 million prescriptions were written for opioids in 2012, enough to give every adult American a bottle of pills [5]. In 2016, more than 11.5 million people reported misuse of prescription pain medication [6] and 115 Americans die every day from an opioid overdose [7]. In 2017, it was estimated that 2 million Americans misused prescription pain medications for the first time which is about 5,480 initiates per day [8]. It has been reported that getting a legitimate prescription during adolescence is associated with a greater risk of opioid misuse especially in young adults with no history of drug use [9]. In 2017, the government declared the opioid crisis a national public health emergency; a declaration which was renewed again in 2018 due to the impact of the opioid epidemic [10].

Arthritis is a chronic disease associated with chronic pain. Patients with rheumatoid arthritis (RA) like other types of arthritis come to seek medical consultation primarily because of their pain [11]. RA patients experience multiple inflammatory and non-inflammatory chronic pain syndrome during their disease course despite the availability of disease-modifying anti-rheumatic drugs (DMARDs) [12]. This warrants the use of different types of very strong pain medications like opioids to alleviate the pain especially after trying less strong options. There are different formulations of opioids that are given for pain which include, immediate release, extended-release, and long-acting with varying duration of action. Osteoarthritis (OA) management is typically focused on symptoms relief and therefore patients are prescribed medications like opioids because there are no disease-modifying regimens currently available for these patients [13]. Unfortunately, with limited options for pain relief after opioids, these patients are at increased risk of drug use disorders. Hence there is the need to explore the trends and factors associated with OUD in hospitalized patients with arthritis.

In this study, we sought to assess the temporal trends of OUD among hospitalized patients with arthritis and identify factors contributing to the increasing trend in OUD during the first five years of the last decade.

## Materials and methods

Study design and data sources

The study was based on the discharge data from the National Inpatient Sample (NIS), Health Care Cost and Utilization Project (HCUP), Agency for Healthcare Research and Quality [14, 15]. This database is the largest all-payer publicly available inpatient care database made up of 20% sample of US hospitalizations, with more than 40 states in the US, the weighted estimate of which represents >95% of all hospitalized US population. We analyzed all adult admissions (18 years and above) from January 1, 2010-December 31, 2014.

International Classification of Diseases Ninth Revision Clinical Modification (ICD-9-CM) was derived from 25-30 diagnoses columns that were used to identify the study population. Quality control procedures performed by the HCUP have demonstrated reliability and accuracy, mainly when data contains the principal diagnosis. Since the database is de-identified and publicly available, ethical clearance or Institutional Review Board approval was not necessary.

Study population and characterization of variables

**Figure 1:**
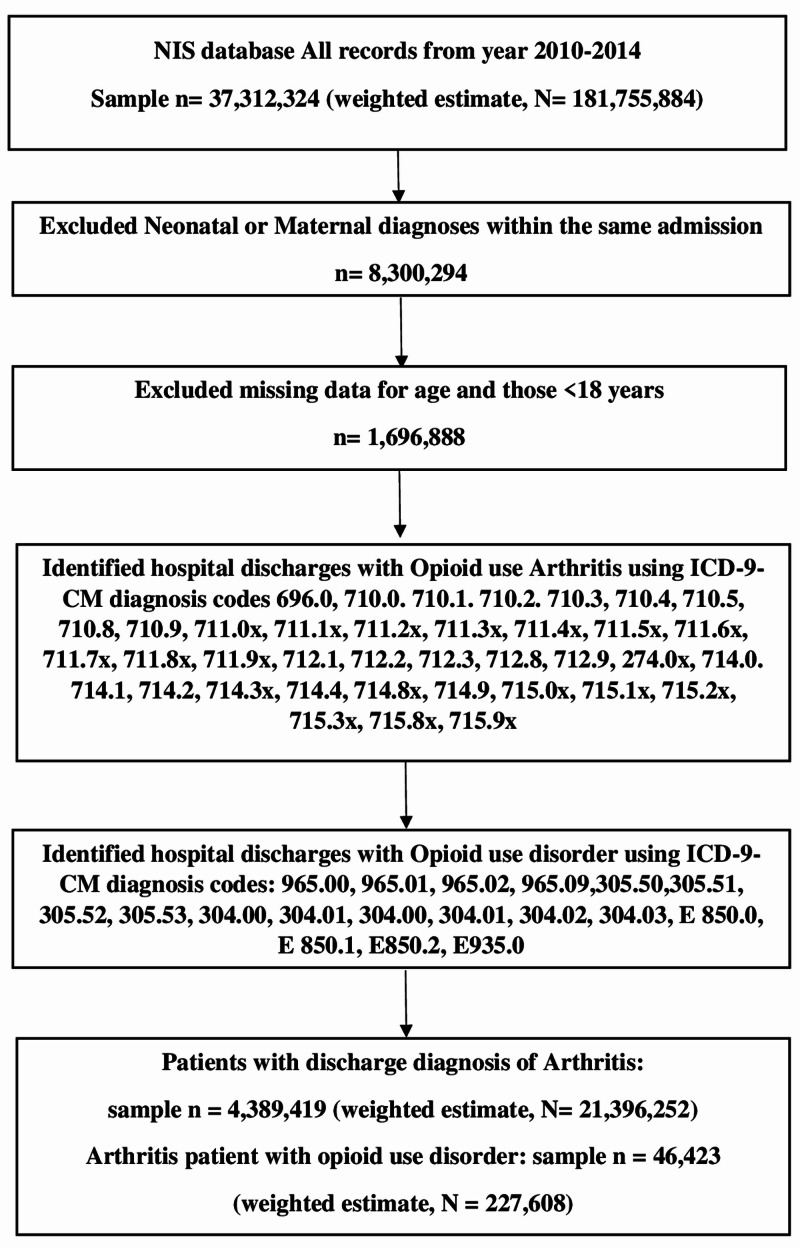
Flowchart for opioid use disorder among arthritis hospitalizations in the United States n, sample number; N, weighted average estimate; ICD-9, International Classification of Diseases 9

The International Classification of Diseases Ninth Revision Clinical Modification (ICD-9-CM) procedure and diagnosis codes were used to identify the diagnosis for OUD in locating the patients of interest in the NIS database. These codes have also been used and verified in previous literature [16]. We also identified ICD-9-CM diagnosis codes for arthritis and the different subtypes of arthritis. The subtypes of arthritis considered were psoriatic arthritis, diffuse disease of connective tissue, infectious arthropathies, crystal arthropathies, rheumatoid arthritis, osteoarthritis, and allied disorders, and other/unspecified arthropathies (Figure 1).

We looked at the trends of OUD among hospitalized patients within these subtypes of arthritis over the five-year study period. We created two groups for comparison among all arthritis hospitalization patients based on the comorbid presence or absence of OUD. We also assessed the demographic and clinical characteristics associated with the increasing trend of OUD among arthritis hospitalization patients.

Patient demographics and comorbidities

Patient-level characteristics from the database included age (sub-divided into 18-24, 25-39, 40-64 and 65 + years), race (white, black and others), the primary payer (government, private, self-pay and others), zip code-based annual median household income (divided into four quartiles), regions of the US (northeast, south, midwest/north-central, and west). Clinical characteristics were derived from the database for patients who had mental health disorders (including anxiety, depression, and psychosis), substance use disorders (including alcohol, marijuana, sedative-hypnotic, cocaine, stimulant, and hallucinogen).

Statistical analysis

STATA version 15.0 (College Station, TX) was used for all statistical analyses. Categorical variables were reported as numbers and percentages while continuous variables were presented as mean and standard deviation. Patients with and without OUD were compared using Student’s t-test and chi-square test where appropriate. We used a P-value of <0.05 and 95% confidence interval (CI). Linear models were used to derive trend analysis using the Joinpoint regression analysis statistical software to derive annual percentage change (APC). The APC considers changes that occur at a constant rate over a specified period in which the rate of disease change is seen in relation to the years assessed in percentages. The Joinpoint software takes trend data and based on the maximum number of Joinpoints supplied by the user, fits the data into segments, enabling the users to assess if the apparent change in trend is statistically significant [17]. All analysis was performed with STRATA and WEIGHT to account for the complex clustered sampling methodology.

## Results

Descriptive characteristics of hospitalized patients with arthritis and OUD

We studied a total of 21,396,252 arthritis hospitalizations identified during the five-year study period from January 1st, 2010 - December 31st, 2014 among which 227,608 (1.1%) had OUD. The patients with arthritis comprised 64.0% female, 77.4% white, and 63.8% aged ≥65 years of hospitalized patients. The overall mean age was 68.8 ± 0.1 years. The demographic and clinical characteristics were compared between OUD and no OUD during arthritis hospitalization (Table 1). Incomes across the quartiles were evenly distributed (28.9% vs 26.7% vs 24.2% and 20.2%). Patients in this population were nearly three times as likely to use government insurance (73.3%) than other types of insurance and the south had more patients on admission than the rest of the regions (39.0%).

**Table 1:** Baseline characteristics of hospitalized patients with arthritis and opioid use disorder n, sample number; N, weighted average; SE, Standard Error; %, Percentage

Name	Overall (n = 4,389,419) (N = 21,396,252)	Opioid use disorder (n = 46,423) (N = 227,608)	No opioid use disorder (n = 4,342,996) (N = 21,168,644)	P-Value
Mean Age (±SE)	68.8 ± 0.1	54.6 ± 0.1	69.0 ± 0.1	<0.0001
Age, years				
18-24	0.4	1.5	0.4	
25-39	2.7	12.5	2.6	
40-64	33.1	63.7	32.8	
≥ 65	63.8	22.3	64.2	<0.0001
Sex, %				
Male	36.0	38.8	36.0	
Female	64.0	61.2	64.0	<0.0001
Race, %				
White	77.4	72.5	77.5	
Black	12.2	16.8	12.2	
Other	10.4	10.7	10.3	<0.0001
Mental health disorders				
Yes	17.3	46.6	16.9	
No	82.7	53.4	83.1	<0.0001
Substance use disorder				
Yes	4.4	38.0	4.0	
No	95.6	62.0	96.0	<0.0001
Obesity				
Yes	17.4	16.5	17.5	
No	80.6	83.5	82.6	<0.0001
Income, %				
First quartile	28.9	35.1	28.8	
Second quartile	26.7	25.9	26.7	
Third quartile	24.2	22.2	24.2	
Fourth quartile	20.2	16.7	20.3	<0.0001
Insurance, %				
Government	73.3	72.8	73.3	
Private	22.5	17.8	22.5	
Self-Pay	1.7	5.5	1.7	
Others	2.5	3.9	2.5	<0.0001
Region, %				
North East	17.1	18.5	17.1	
Mid-West/North Central	26.7	22.0	26.8	
South	39.0	34.4	39.0	
West	17.2	25.1	17.2	<0.0001
Hospital Teaching Status, %				
Rural	13.1	8.8	13.1	
Urban non-teaching	39.9	38.7	39.9	
Urban teaching	47.0	52.5	47.0	<0.0001
Discharge disposition, %				
Home/Home health	71.9	78.1	71.9	
Others	28.1	21.9	28.1	<0.0001
Length of stay, days	4.7 ± 0.02	5.8 ± 0.05	4.7 ± 0.02	<0.0001
Cost (± SE), $	13270.82 ± 86.67	11603.74 ± 144.83	13288.78 ± 86.75	<0.0001
Mortality, %	1.5	0.9	1.6	<0.0001

In the overall arthritis population, osteoarthritis makes up the highest number of hospitalized arthritis patients (68.0%) followed by unspecified arthropathies (14.3%) and rheumatoid arthritis (9.4%). However, within the arthritis groups, the proportion of those who had OUD was highest among the infectious arthritis group (3.7%) followed by psoriatic arthritis (2.2%) and diffuse disease of connective tissue (1.9%) (Table 2).

**Table 2:** Types of hospitalized arthritis patients with opioid use disorder PA, Psoriatic arthritis; Diffuse CT, Diffuse disease of connective tissue; IA, Infectious arthropathies; CA, Crystal arthropathies; RA, Rheumatoid arthritis; OA, Osteoarthritis and allied disorders; UA, Other/unspecified arthropathies; %, percentage

Opioid use disorder	PA	Diffuse CT	IA	CA	RA	OA	UA
Total (%)	19,288 (0.5%)	194,265 (4.4%)	52,390 (1.2%)	97,905 (2.2%)	412,102 (9.4%)	2,984,705 (68.0%)	628,764 (14.3%)
Opioid use disorder (%)	416 (2.2%)	3,698 (1.9%)	1,929 (3.7%)	708 (0.7%)	6,817 (1.7%)	24,649 (0.8%)	8,206 (1.3%)

Trends and predictors of hospitalizations with arthritis and OUD

**Figure 2:**
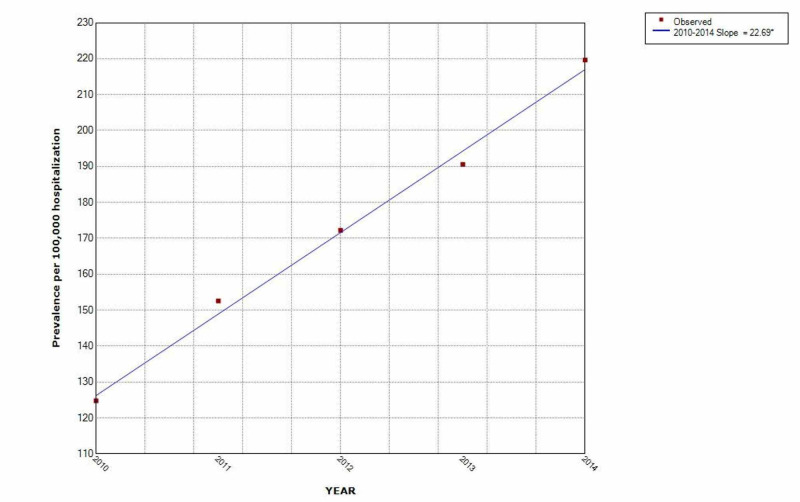
Increasing trend of opioid use disorder among arthritis hospitalization over the five-year study period * indicates that the slope is significantly different from zero at the alpha = 0.05 level. Final selected model: 0 Joinpoints

The prevalence of OUD in arthritis hospitalization increased over the five-year period by 43% (Figure 2). After adjusting for other variables, multivariate logistic regression [OR (95% CI), p < 0.001] determined the following were associated with increased odds of opioid use disorder: age group 25-39 years (OR 1.12 (1.02-1.24)) compared to 18-24 years; females (OR 1.07 (1.04-1.10)); hospitals in US west region (OR 1.48 (1.38-1.59)) compared to Northeast region; urban teaching hospitals (OR 1.41 (1.30-1.54)) and urban non-teaching hospitals (OR 1.36 (1.25-1.48)) compared to rural hospitals (Table 3).

**Table 3:** Factors associated with opioid use disorder in hospitalized patients with arthritis SE, Standard error; %, percentage; OR, Odds ratio; Ref, Reference

Name	Univariate analysis (Crude OR)	P-Value	Multivariate analysis (Adjusted OR)	P-Value
Mean Age (±SE)	0.94 (0.94-0.94)	<0.0001		
Age, years				
18-24	Ref			
25-39	1.35 (1.24-1.48)	<0.0001	1.12 (1.02-1.24)	0.0210
40-64	0.54 (0.50-0.60)	<0.0001	0.60 (1.28-1.44)	<0.0001
≥65	0.10 (0.09-0.11)	<0.0001	0.12 (0.11-0.13)	<0.0001
Sex, %				
Male	Ref			
Female	0.89 (0.86-0.91)	<0.0001	1.07 (1.04-1.10)	<0.0001
Race, %				
White	Ref			
Black	1.47 (1.36-1.58)	<0.0001	0.90 (0.84-0.97)	0.0030
Other	1.10 (1.04-1.18)	<0.0020	0.78 (0.74-0.82)	<0.0001
Mental health disorder				
No	Ref			
Yes	4.28 (4.16-4.41)	<0.0001	2.50 (2.43-2.58)	<0.0001
Substance use disorder				
No	Ref			
Yes	14.68 (14.18-15.20)	<0.0001	6.39 (6.14-6.66)	<0.0001
Obesity				
No	Ref			
Yes	0.94 (0.91-0.97)	<0.0001	0.81 (0.78-0.84)	<0.0001
Income, %				
First quartile	Ref			
Second quartile	0.79 (0.76-0.83)	<0.0001	0.91 (0.88-0.95)	<0.0001
Third quartile	0.75 (0.72-0.79)	<0.0001	0.89 (0.86-0.93)	<0.0001
Fourth quartile	0.68 (0.64-0.72)	<0.0001	0.87 (0.83-0.92)	<0.0001
Insurance, %				
Government	Ref			
Private	0.80 (0.77-0.83)	<0.0001	0.43 (0.42-0.45)	<0.0001
Self- Pay	3.33 (3.08-3.60)	<0.0001	0.92 (0.85-0.99)	0.0190
Others	1.57 (1.46-1.69)	<0.0001	0.65 (0.60-0.69)	<0.0001
Region, %				
North East	Ref			
Mid-West/North Central	0.76 (0.69-0.83)	<0.0001	0.80 (0.73-0.87)	<0.0001
South	0.81 (0.75-0.89)	<0.0001	0.83 (0.78-0.89)	<0.0001
West	1.35 (1.24-1.46)	<0.0001	1.48 (1.38-1.59)	<0.0001
Hospital Teaching Status, %				
Rural	Ref			
Urban non-teaching	1.44 (1.32-1.56)	<0.0001	1.36 (1.25-1.48)	<0.0001
Urban teaching	1.66 (1.53-1.80)	<0.0001	1.41 (1.30-1.54)	<0.0001
Length of stay, days	1.02 (1.02-1.02)	<0.0001	1.01 (1.01-1.01)	<0.0001
≥Mortality, %	0.01 (0.01-0.01)	<0.0001	0.81 (0.73-0.90)	<0.0001

However, the following were associated with reduced odds of OUD: age group 40-64 years (OR 1.12 (1.02-1.24)) and ≥65 years (OR 0.12 (0.11-0.13)) compared to 18-24 years; blacks (OR 0.90 (0.84-0.97)) compared to whites; those who are obese (OR 0.81 (0.78-0.84)) compared to non-obese; higher-income quartile e.g. second income quartile (OR 0.91 (0.88-0.95)) compared to lowest income quartile.

Substance use and mental health disorders as associated factors of OUD in arthritis hospitalizations

The proportions of patients hospitalized for arthritis who also had mental health and substance use disorders were 17.3% and 4.4%, respectively. The identified mental illness disorders in the arthritis patients were mood disorders (4.5%), anxiety (13.9%), and psychosis (0.1%). While the identified substance of abuse in those with substance use disorders in the arthritis patients were marijuana (0.6%), alcohol (2.9%), cocaine (0.5%), stimulant (0.1%), and sedative-hypnotic (0.4%). After adjusting for other variables, mental health [OR 2.50 (2.43-2.58)], and substance use [OR 6.39 (6.14-6.66)] disorders were associated with increased odds of OUD.

## Discussion

This study showed that the prevalence of opioid overdose among hospitalized arthritis patients is on the rise. The prevalence increased by 43% in the five-year study period. To the best of our knowledge, this is the first study describing the trend of OUD in hospitalized arthritis patients. The odds of OUD are higher in in-hospital patients aged 25-39 years, females, hospitals in the US west region, urban teaching, and non-teaching hospitals. In our study ages 25-39 years were more vulnerable to OUD compared to other age groups with a monographic decline as the age progresses. These findings support previous studies that found that young adults are at increased risk of opioid overdose in spinal conditions compared to other age groups [16].

In part, due to the failure of DMARDS to treat the chronic pain of arthritis patients effectively, the number of arthritis patients being given opioids has increased [18]. Also, the nonpharmacological treatment options for arthritis have been studied in recent times, with a meta-analysis conducted by Corbett et al. questioning the efficacy of these treatment options for arthritis [19]. Such treatments include acupuncture, pulsed electrical stimulation, balneotherapy, aerobic exercise, and muscle-strengthening exercise. About a third of RA patients are now being prescribed opioids and this prediction is said to continue to increase in the coming years in an already opioid-burdened population [1]. All pain is not the same, just as all modalities of pain control are not the same. For example, there are different modalities of giving opioids which include, patient-controlled analgesia (PCA), intrathecal, and epidural analgesia [20]. Despite the availability of opioids for pain relief especially in the last few decades, there are still no strong recommendations in the medical community for or against the use of opioids in the treatment of pain for all forms of arthritis [21-23].

Our study found a reduced risk of OUD in arthritis patients who are obese. However, Stokes et al. reported the reverse where obesity and OUD were found to be significantly associated as body mass index (BMI) rises [24]. However, there is a possibility that those who were obese and had the same dose of opioid as those who were not obese may not turn out to be at reduced risk of OUD because of their higher body mass index which may be protective due to their drug pharmacodynamics. It may be that a similar dose has no adverse effect of OUD in the obese than in the non-obese. Also, if our study groups were divided into long- and short-term opioid use, our findings may be different after stratification of opioid use. Studies have shown that long-duration use of opioids is a major risk factor for OUD [25]. Obesity has been associated with increased risk of chronic conditions including osteoarthritis and low back pain leading to the rise in prevalence of chronic pain and hence the need for long term opioid use [24, 26, 27].

The greatest predictors of OUD in our study were patient-specific comorbidities. These comorbidities were substance use disorders and mental health disorders. Our findings of an association between substance use disorder and an increased risk of OUD agree with the findings of previous studies [28]. This is consistent with a study that found that patients with ankylosing spondylitis (AS) taking opioids medication were more likely to use psychoactive medication like sedatives [29]. Previous studies have also reported that substance use disorders and mental health conditions contribute to the chronic use of opioid in hospitalized patients [30].

There have been proposals to decrease opioid overdose at all levels both at institutional and provider levels [28]. This study adds to the awareness of the problem and targets intervention to patients at greatest risk. Predictors such as mental health disorders and substance use disorders can be used to implement institutional level changes. Such changes may include the use of electronic medical records to alert their users to administer opioids with caution to patients who are vulnerable. Providers can be educated on the use of opioids through safer and alternative delivery methods such as advocacy of exercise and weight control.

The limitations of this study include the source of the database. For example, the NIS database is an administrative database that is prone to coding errors such as incorrect coding or missing data from inconsistent coding practices. Critical data can be underreported in some cases making the data less accurate. Also, the database might be less accurate in reporting the number of arthritis patients, given that there may be other comorbidities that may account for patient's hospitalization other than arthritis. Another limitation is the inability of the NIS database to evaluate opioid use patterns and dosage which may help predict why a patient may be at risk of OUD. However, the strength of this study is the availability of a large sample size and a longitudinal analytical database with clinical and demographic data across the United States. This large database increases study power and generalizability of findings.

## Conclusions

Our study showed that the prevalence of OUD among patients with arthritis is on the rise and mental health and substance use disorders were associated with increased odds of opioid use disorder. More studies are needed to explore alternative pain management therapies for arthritis patients particularly in those with mental health and substance use disorders to improve their quality of life while reducing the burden of chronic opioid therapy.

## Appendices

**Table 4:** International Classification of Diseases-9 codes used for mental health and substance use disorder ICD-9, International Classification of Diseases-9

Diagnosis	ICD-9 code
Alcohol	303.00, 303.01, 303.02, 303.03, 303.90, 303.91, 303.92, 303.93, 305.00, 305.01, 305.02, 305.03, 980.0, 980.1, 980.2, 980.3, 980.8, 980.9
Marijuana	304.30, 304.31, 304.33, 305.20, 305.21, 305.22, 305.23
Sedative-hypnotic	304.10, 304.11, 304.13, 305.40, 305.41, 305.42, 305.43, 967.0, 967.1, 967.2, 967.3, 967.4, 967.5, 967.6, 967.8, 967.9, 969.4, E851, E852.0, E852.1, E852.2, E852.3, E852.4, E852.5, E852.8, E852.9, , E853.0, E853.1, E853.2, E853.8, E853.9, E937.0, E937.1, E937.2, E937.3, E937.4, E937.4, E937.5, E937.6, E937.7, E937.8, E937.9
Cocaine	304.20, 304.22, 304.23, 305.60, 305.61, 305.62, 305.63, 970.81, 970.89
Stimulant	304.00, 304.41, 304.42, 304.43, 305.70, 305.71, 305.72, 305.73, 969.6, 970.0, 970.1, 970.9, E854.2, E854.3, E854.9
Hallucinogen	304.50, 304.51, 304.52, 304.53, 305.30, 305.31, 305.32, 305.53, E854.1, E855.5, E855.6, E855.8, E855.9
Others substance use	304.60, 304.61, 304.62, 304.63, 304.70, 304.71, 304.72, 304.73, 304.80, 304.81, 304.83, 304.90, 304.91, 304.92, 304.93, 305.80, 305.81, 305.82, 305.83, 305.90, 305.91, 305.92, 305.93, 969.0, E939.0, E939.1, E939.2, E939.2, E939.3, E939.4, E939.5, E939.6, E939.6, E939.7, E939.8, E939.9
Mood disorder	293.83, 296.00, 296.01, 296.02, 296.03, 296.04, 296.05, 296.06, 296.10, 296.11, 296.12, 296.13, 296.14, 296.15, 296.16, 296.20, 296.21, 296.22, 296.23, 296.24, 296.25, 296.26, 296.30, 296.31, 296.32, 296.33, 296.34, 296.35, 296.36, 296.40, 296.41, 296.42, 296.43, 296.44, 296.45, 296.46, 296.50, 296.51, 296.52, 296.53, 296.54, 296.55, 296.56, 296.60, 296.61, 296.62, 296.63, 296.64, 296.65, 296.66, 296.7, 296.80, 296.81, 296.82, 296.89, 296.90, 296.99, 300.4, 311
Anxiety	293.84, 300.00, 300.01, 300.02, 300.09, 300.10, 300.20, 300.21, 300.22, 300.23, 300.29, 300.3, 300.5, 300.89, 300.9, 308.0, 308.1, 308.2, 308.3, 308.4, 308.9, 309.81, 313.0, 313.1, 313.21, 313.22, 313.3, 313.82, 313.83
Psychosis	297.0, 297.1, 297.2, 297.3, 297.8, 297.9, 298.0, 298.1, 298.2, 298.3, 298.4, 298.8

**Table 5:** ICD-9 codes used for arthritis subtypes and obesity ICD-9, International Classification of Diseases 9

Diagnosis	ICD-9 code
Psoriatic arthritis	696.0
Diffuse disease of connective tissue	710.0. 710.1. 710.2. 710.3, 710.4, 710.5, 710.8, 710.9
Infectious arthropathies	711.0x, 711.1x, 711.2x, 711.3x, 711.4x, 711.5x, 711.6x, 711.7x, 711.8x, 711.9x
Crystal arthropathies	712.1, 712.2, 712.3, 712.8, 712.9, 274.0x
Rheumatoid arthritis	714.0. 714.1, 714.2, 714.3x, 714.4, 714.8x, 714.9,
Osteoarthritis and allied disorders	715.0x, 715.1x, 715.2x, 715.3x, 715.8x, 715.9x
Other/unspecified arthropathies	716.0x, 716.1x, 716.2x, 716.3x, 716.5x, 716.6x, 716.8x, 716.9x
Obesity	278.00, 278.01, 278.03
